# A retrospective analysis of health-related quality of life in adolescent idiopathic scoliosis children treated by anterior instrumentation and fusion

**DOI:** 10.1186/s13013-018-0162-2

**Published:** 2018-08-30

**Authors:** Balaji Zacharia, Dhiyaneswaran Subramaniyam, Sadiqueali Padinharepeediyekkal

**Affiliations:** Department of Orthopedics, Government Medical College, Kozhikode, Kerala 673008 India

**Keywords:** Health-related quality of life, Adolescent idiopathic scoliosis, Anterior instrumentation and fusion, SRS-30

## Abstract

**Background:**

Idiopathic scoliosis is the most common type of spinal deformity. Scoliosis is defined as a lateral curvature of the spine greater than 10° accompanied by rotation of the vertebrae. The treatment available for adolescent idiopathic scoliosis is observation, orthosis, and surgery. The surgical options include open anterior release and instrumentation, posterior instrumentation, and thoracoscopic approaches. The Scoliosis Research Society Questionnaire (SRS-30) is a specific instrument to measure health-related quality of life in patients with scoliosis, who had or had not undergone surgery. The purpose was to assess the post-operative functional outcome using SRS-30 in children who underwent anterior release, instrumentation, and fusion using autogenous rib graft for adolescent idiopathic scoliosis (AIS).

**Methods:**

In a retrospective cohort study, 25 patients between the ages of 11 and 17 years, who underwent anterior release, instrumentation, and fusion using autogenous rib graft for adolescent idiopathic scoliosis (AIS) between 2008 and 2014, were included in the study.

**Results:**

The total average score was 4.26 with a SD of 0.014 and had maximum average score 4.5 (for pain) and minimum average score 3.8 (for self-image).

**Conclusion:**

Anterior release, instrumentation, and fusion using autogenous rib graft is having good functional outcome in all domains.

## Background

Idiopathic scoliosis is the most common type of spinal deformity attended by orthopedic surgeons [1]. Scoliosis is defined as a lateral curvature of the spine greater than 10° accompanied by rotation of vertebrae [2].The prevalence of radiographic curve measuring up to 10° ranges from 1.5 to 3% and that of the curve exceeding 10° is from 0.3 to 0.5%. The prevalence of the curve exceeding 30° is 0.2 to 0.3% [3]. For curves less than 10°, there is an equal prevalence in both sexes, but the higher the degree of curves, the more is the prevalence in females [4, 5]. The etiology of idiopathic scoliosis remains unknown. There are many proposed etiological factors like genetic and neurological disorders, hormonal and metabolic dysfunction, and biomechanical and environmental factors [6].

In structural scoliosis, the vertebral body is rotated towards the convex side of the curve, so the spinous process is rotated towards the concave side of the curve. The asymmetric deformities seen within the vertebral bodies of scoliosis differ significantly from its normal counterpart. The compressive and the distractive force acting on the growing spine produces wedging of the vertebrae. The rotation of the vertebrae produces a hypokyphotic or lordotic curvature of the spine in the sagittal plane. This three-dimensional deformity is better termed as the torsion of the spine and is maximum at the apex of the curve [7–9]. The aorta is usually positioned more laterally and posteriorly in idiopathic adolescent scoliosis [10]. Thoracic cavity is asymmetrical in shape with increased capacity on the concave side and decreased on the convex side. There will be rib prominence on the convex side-rib hump, and breast on the concave side will be more prominent.

There is no definite definition regarding curve progression in the literature but an increase of more than 5–6° over 6 months is considered to be an indicator of progression by most of the studies. Factors such as family history of scoliosis, patient’s height to weight ratio, thoracic kyphosis, lumbosacral transition anomaly, spinal balance, and lumbar lordosis are having high predictive value for curve progression in skeletally immature patients [11–13].

Curves less than 40° at maturity may progress to an average of 9° during adulthood and curves more than 40° progress to an average of 20° [14]. Adolescent idiopathic curve does not produce long-term pulmonary functional abnormalities, even though it can produce some restrictive disease. But untreated infantile and juvenile curves and severely lordotic thoracic curves of high degree can result in pulmonary problems [15]. In about 32% of AIS, back pain is reported with increased incidence towards maturity. There is no association between the degree of curvature and back pain [16].

There are different systems to classify scoliosis like Ponseti and Fredman classification, King Classification, and Lenke classification. Lenke is the most recent and commonly used classification system. There are three steps involved in this classification system: identification of the primary curve, assignment of the lumbar modifier, and assignment of the thoracic sagittal modifier [17–19].

The treatment options available for adolescent idiopathic scoliosis are observation, non-surgical intervention, and surgical intervention. In general, no treatment is needed for curves less than 25° regardless of the patient maturity, but follow-up examinations are necessary at regular intervals. Bracing is recommended for skeletally immature patients having curves between 25° and 45° [20, 21]. Numerous authors have challenged the effectiveness of bracing for AIS [22, 23]. The BRAIST study found that bracing significantly reduces the progression of high-risk curve to the threshold for surgery [24].

In skeletally immature patients in whom the curve reaches 40° to 50°, or skeletally mature patients with curves greater than 50°, surgery is a reasonable option [6]. The primary aim of surgical intervention is to reduce the magnitude of the deformity and to obtain solid fusion for prevention of further progression of curve. It results in a well-balanced spine in which head, shoulders, and trunk are centered over the pelvis. Open posterior instrumentation and fusion, open anterior instrumentation and fusion, and thoracoscopic techniques are used for achieving solid arthrodesis and obtaining a balanced three-dimensional correction of the spine. The primary concern of posterior instrumentation in very young patients is the occurrence of crankshaft phenomenon [25]. The risk of crankshaft phenomenon with modern posterior pedicle screw instrumentation has been very minimal in the recent years [26]. Therefore, the anterior surgery for prevention of crankshaft has got limited role in recent years.

The correction and stabilization for scoliosis through anterior approach is introduced by Dwyer in 1964 [27]. Later several modifications of the Dwyer system were done like the Zidelke system (1970), TSRH system (1980), dual-rod dual-screw technique (1990), and L plate system (2006). The advantages of anterior correction include fewer number of fusion levels, excellent correction of deformity, maintaining dorsal kyphosis, implants not prominent under the skin, and preventing crankshaft phenomenon. There is a cosmetic advantage of having smaller scar compared to the posterior approach. However, its limitations are reduction of segmental lumbar lordosis and substantial pseudarthrosis [28]. The indications for anterior fixation are single structural thoracic or thoracolumbar curve (Lenke types I and V), curves less than 70°, and severe thoracic hypokyphosis. It is contraindicated in patients with pulmonary compromise, severe intra-thoracic scarring, small patient size, and severe osteopenia.

Patient-reported health-related quality of life (HRQOL) outcome questionnaires have gained popularity as the method to objectively assess baseline pathology and to measure the effectiveness of an intervention [29]. The Scoliosis Research Society Questionnaire (SRS-30) is a specific instrument to measure health-related quality of life in patients with scoliosis who had undergone surgery or had not [30]. The SRS-30 is well-known as a standard assessment tool to evaluate patients’ quality of life across five domains: function/activity, pain, self-image/appearance, mental health, and satisfaction with management [31]. It was Haher [32] who initiated the development of the disease-specific HRQOL instrument SRS-24,consisting of 24 items, to measure many aspects of spinal deformity. Later in 2000, Asher et al. merged similar domains and the new questionnaire SRS-23 was formed [33]. In 2006, Asher et al. again refined the questionnaire to address the diminishing of internal consistency for the function domain in adolescent idiopathic scoliosis [34]. Various versions, scoring instruction, and detailed bibliography of the development of the SRS-HRQOL can be found on the scoliosis research society website www.srs.org [29].

We have conducted a retrospective cohort study to assess the functional outcome of adolescent idiopathic scoliosis (Lenke types I and V) patients treated by anterior release, instrumentation, and fusion using autogenous rib graft.

## Methods

After obtaining institutional research committee and ethics committee approval of Government Medical College Kozhikode [Ref no: GMCKKD/RP 2014/IEC/34/03 dtd 17 March 2014], we conducted a retrospective cohort study in a consecutive series of children with adolescent idiopathic scoliosis who underwent anterior release, instrumentation, and fusion using autogenous rib graft in the orthopedic department of our institution. The primary indication for surgery was cosmetic correction of the deformity. There were no cases with back pain. All patients included in the study were concerned about the physical appearance of the back and that was the main indication for surgery. They were included after obtaining the written informed consent from the parents. There were 30 patients who underwent surgical treatment during the period from August 2008 to December 2014. Out of the 30 patients, one patient expired due to unrelated problem and we lost follow-up of four patients. The remaining 25 patients included in the study were having either type I or type V curve after obtaining informed consent from their parents. No children with thoracic insufficiency syndrome was included in our study, and those who underwent posterior procedure were excluded.

All surgery were done under general anesthesia, and preoperative prophylactic antibiotics were given. The patients were positioned laterally with convex side up and thoracotomy/thoracolumbar approach was used. Exposure was completed by removing the rib of one level above the most proximal instrumented vertebrae. After exposing the vertebrae, the anterior release was done by removing disc material and endplate. Correction was done by derotating the apical vertebrae and fixation using pedicle screw and rod instrumentation. Fusion was aided with the help of autogenous rib graft. The whole procedure was done under motor-evoked potential surveillance. Suture removal was done on the 14th day, and patients were discharged with thoracolumbosacral orthosis, which they wore till complete fusion, that was achieved in 9 months.

The age of patients at the time of final evaluation was between 11 and 17 years. SRS-30 was used to measure the patient outcome at the time of the final follow-up. It was divided into five domains according to question type—pain, function /activity, self-image/appearance, mental health, and satisfaction with management. All questions had scores from 1 to 5. The mean score of each domain was used for analysis. The best score is 5 and the worst score is 1.

## Results

**Fig. 1:**
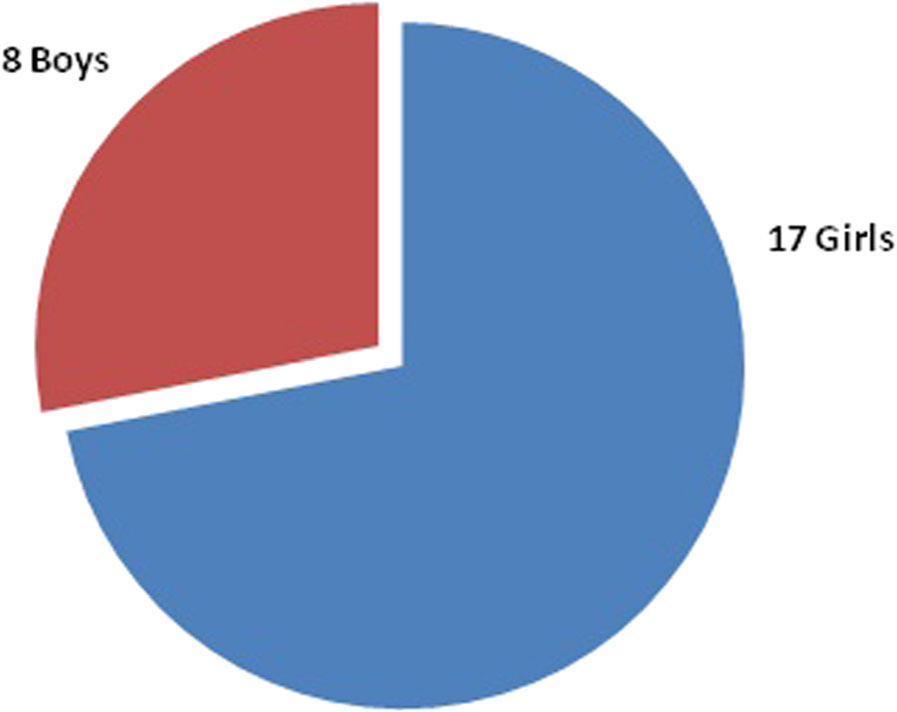
Sex distribution of children

**Fig. 2:**
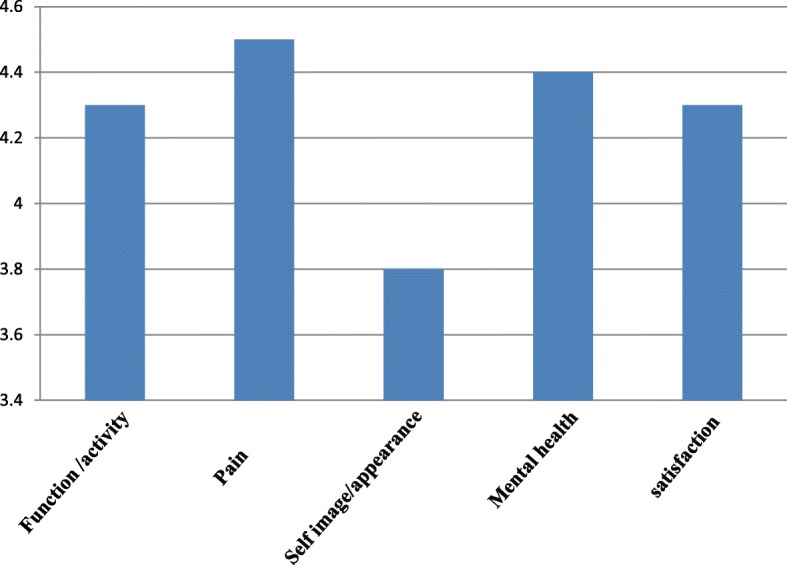
Average SRS-30 score in each domain

Our study included 17 girls and 8 boys (Fig. 1). Average duration of follow-up was 4 years and 4 months with SD of 2 years and 3 months [4.3 ± 2.25] (Table 1). There were 13 children with a pre-operative Cobb angle between 45° and 50°, 9 patients with an angle 50°–60°, 2 patients with an angle between 60° and 65°, and 1 patient with a Cobb angle of above 70° (72°). Post operatively, 21 patients were having a Cobb angle of less than 20° and remaining patients had between 20° and 25°. Out of the five domains of SRS-30, we got maximum average score 4.5 for pain domain and minimum 3.8 for self-image/appearance. For functional activity domain, average score was 4.3. The average scores for mental health and satisfaction with management were 4.4 and 4.3 respectively (Fig. 2). The total average score was 4.26 with a SD of 0.014 (Table 2).

**Table 1:** Case follow-up

Year of surgery	Number of cases	Duration in years at final follow-up after surgery
2008	4	7
2009	3	6
2010	4	5
2011	3	4
2012	4	3
2013	4	2
2014	3	1

**Table 2:** SRS -30 data

Domain	Average score	Standard deviation (SD)	Maximum	Minimum
Function/activity	4.3	0.18	4.6	4.0
Pain	4.5	0.18	4.9	4.3
Self-image/appearance	3.8	0.2	4.2	3.4
Mental health	4.4	0.3	5.0	3.8
Satisfaction with management	4.3	0.3	4.8	3.5
Total	4.26	0.014	4.5	4.0

## Discussion

In this study, we retrospectively analyzed functional outcome of 25 patients with adolescent idiopathic scoliosis, who underwent anterior release, instrumentation, and fusion using autogenous rib graft. All the surgeries were done by a single surgeon. We got grade 1 fusion in 84% of the cases, grade 2 in 12% of the cases, and grade 3 in 4% of the cases, using Newton et al. grading system for spinal fusion [35]. No case of pseudarthrosis or instrumentation failure was detected in our study. Currently, there is an increasing awareness among orthopedic surgeons for evaluating functional over the radiological outcome. There are three types of outcome measures used in orthopedic surgery generic, disease-specific, and anatomy- or joint-specific outcome measures. These outcome measures must have appropriate content for relevant disease or process, reproducibility of results, and responsiveness which is sufficient to detect clinically important change and ease of use for clinicians and patients [36]. In 2013, Sudo et al. conducted a study on the long-term outcome of anterior correction of scoliosis. Their average total SRS score was 4.2 [37]. A study by Sweet et al. in 2001 came to the conclusion that anterior instrumentation and fusion for AIS using a single solid rod has good radiological and clinical outcomes. Poor radiological outcome did not correlate with final Scoliosis Research Society score [38]. Kelly et al. reported an absolute SRS score of 98 of 115 points without much postoperative complication on an average follow-up of 16.97 years [39]. After evaluating the clinical outcome of anterior endoscopic instrumentation for scoliosis, using SRS-24 showed a significant improvement in pain, self-image, and function in a 2-year follow-up period [40]. In a study by Chan et al., using the results of SRS-22 and SRS-24, to compare the clinical results of various treatment methods, the conclusion was that the values of the scores of these two questionnaires cannot be used interchangeably despite the similarities in the questions and domains [41].

The results of our study cannot be compared with other similar studies conducted to find out HRQL of children with adolescent idiopathic scoliosis treated by using braces and corrective surgeries through anterior or posterior approach because of sociocultural difference among different population. The sociocultural differences will affect the domain of SRS. We have no such similar studies from our population for comparison. This is the first ever attempt to find out HRQL of adolescent idiopathic scoliosis children post-operatively from an Indian subcontinent.

Our study has several limitations. We have conducted this study in a small number of patients. The follow-up of patients varied from 1 to 7 years. We did not have a preoperative patient-based questionnaire, so longitudinal analysis of this measure was not possible.

## Conclusion

From our study, using the SRS-30 scoring system in Lenke I and V, we found that anterior release, instrumentation, and fusion using autogenous rib graft is having good functional outcome in all domains: function, pain, self-image, mental health, and satisfaction with management.

## Abbreviations

AIS: Adolescent idiopathic scoliosis
HRQOL: Health-related quality of life
